# Incidental Detection of an Extra-abdominal Desmoid Tumor of the Axilla: A Case Report

**DOI:** 10.7759/cureus.109162

**Published:** 2026-05-19

**Authors:** Chalent Alexakis, Annezo Marinatou, Evgenia Kalogridaki, Nektarios Ntalakos, Panagiotis Daskalakis

**Affiliations:** 1 Rural Medicine, General Hospital of Thebes, Thebes, GRC; 2 Surgery Department, Breast Unit, General Maternal Hospital of Athens ‘Elena Venizelou’, Athens, GRC; 3 Pathology, Saint Savvas Anticancer Hospital of Athens, Athens, GRC

**Keywords:** benign breast lesion, breast surgery, extra-abdominal desmoid tumor, fibromatosis, incidental discovery

## Abstract

Desmoid fibromatosis is a rare, benign but aggressive tumor of mesenchymal origin. Its main characteristic is local infiltration and a high likelihood of recurrence. Desmoid tumors can occur either sporadically or in a familial form (familial adenomatous polyposis (FAP)). Depending on their location, they are classified as superficial or deep, such as masses in the abdominal wall, extra-abdominal, or intra-abdominal tumors. Rarely, they can be found in the axillary area. We describe a case of a 69-year-old female presenting with a mass in her left axilla that was biopsy-proven to be a desmoid tumor.

## Introduction

Desmoid tumors are very rare and develop more frequently in patients with familial adenomatous polyposis (FAP) (Gardner syndrome). They have a benign histological appearance and do not give distant metastases, but they have the tendency not only to expand locally and infiltrate important anatomical structures but also to recur frequently after removal [1]. The male-to-female ratio in desmoid tumors indicates that women are more commonly affected than men. Although the ratio varies from study to study, it is typically considered to be between 1:2 and 1:3 [2]. Some studies have found a link between estrogen levels and this female predominance, suggesting that hormonal mechanisms may be involved. It occurs infrequently, with an incidence of approximately 0.03% of all tumors [2].

With a high occurrence between the ages of 30 and 40, desmoid tumors manifest as a large palpable mass in women aged 15 to 60 [1,3]. Histologically, desmoid tumors are defined by the proliferation of homogenous spindle-shaped cells resembling myofibroblasts against a background of rich collagenous tissue. The characteristic that helps differentiate them from other soft tissue lesions is the prominent vascular structures that may be observed, which contribute to the tumor's vascularity [4]. The proliferation of homogeneous spindle-shaped cells that resemble myofibroblasts against a background of rich collagenous tissue is a histological characteristic of a desmoid tumor. A network of blood arteries is frequently present in the tumor, adding to its vascularity. This specific feature is important for the distinction between desmoid tumors and other soft tissue masses [4]. Typically, there are no specific imaging findings, but due to their rapid growth, they may appear as a large, well-defined mass in the breast, which can sometimes show signs of ossification [5]. Due to the heterogeneity and rarity of these tumors, there is insufficient information regarding their clinical characteristics and prognosis [6].

The current report aims to present a rare case of extra-abdominal desmoid tumor of the left axilla.

## Case presentation

A 69-year-old female patient presented to our clinic with a self-detected left breast lump. There were no macroscopic changes in the shape or color of the breast and nipple. Her past medical history was unremarkable. Her past surgical history includes a thyroidectomy at the age of 64. She is a non-smoker and does not consume alcohol. Her family history is free of any significant pathologies. On examination, there was a palpable mass in the lower outer quadrant, and the examination of the contralateral breast revealed no abnormalities. Lymphadenopathy was not detected in either axilla. Mammography revealed a radiopaque oval-shaped lesion with partially indistinct margins at the junction of the outer quadrants (posterior third). The ultrasound revealed a cyst at the border of the eighth to ninth hour of the left breast, measuring 11 x 10 x 6 mm. Color Doppler imaging did not demonstrate significant vascularization. A fine-needle aspiration (FNA) was performed, and the patient was advised to undergo a follow-up ultrasound in three months. The results of the FNA revealed no findings suggestive of malignancy, but the patient did not return for the follow-up ultrasound. After one year, the patient returned for her annual breast screening, complaining that she was unable to fully lower her left arm due to the presence of a mass in the axillary region. The mammogram did not reveal any abnormalities; however, the ultrasound detected multiple confluent masses resembling lymph nodes in the left axillary region with a rounded shape, low echogenicity of the parenchyma, and no visible hilum, measuring up to 38 mm in diameter. On clinical examination, lymph nodes were palpated in the axillary region, and restricted arm adduction was observed. It was advised that the patient undergo a breast MRI and follow-up. The imaging test revealed the presence of a markedly enlarged block of left axillary lymph nodes, extending over an area of approximately 8.2 × 10.6 cm in the transverse plane (Figure 1), compressing surrounding structures (Figure 2). Clinical correlation and mandatory histological confirmation were required.

**Figure 1:**
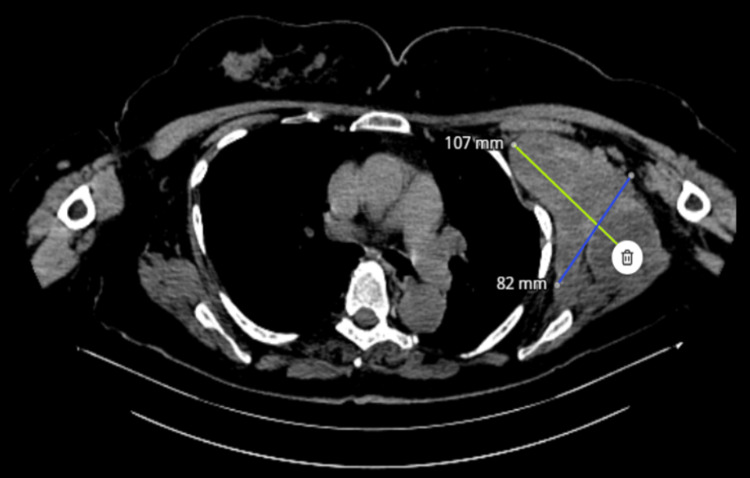
Axial CT image of the thoracic region showing the desmoid tumor in the left axilla.

**Figure 2:**
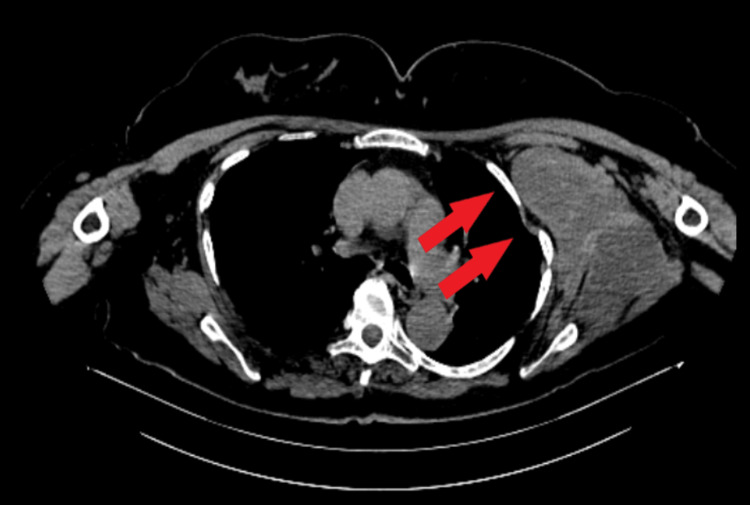
Axial CT image of the thoracic region showing a tumor squeezing the thoracic cage (the locations of compression are shown by the red arrows).

The patient was scheduled for a core biopsy of the left axillary region for further histopathological evaluation. The results showed fragments of fibro-fatty tissue, within which scattered, widely confluent tissue structures were observed, resembling necrobiotic parasitic forms. Histochemical staining was performed with PAS (-), Giemsa (-), and Ziehl-Neelsen (-). The biopsy slides were sent to a hospital with a specialized histopathological department for further evaluation, which revealed a mesenchymal tumor. Before surgery, the patient underwent chest and upper and lower abdomen computed tomography to assess for metastases, which showed no distant spread of the disease. The patient then underwent surgical excision of the tumor. Given the extension into the surrounding structures like the heart and thoracic cage, only a partial resection was able to be accomplished (Figures 3, 4).

**Figure 3:**
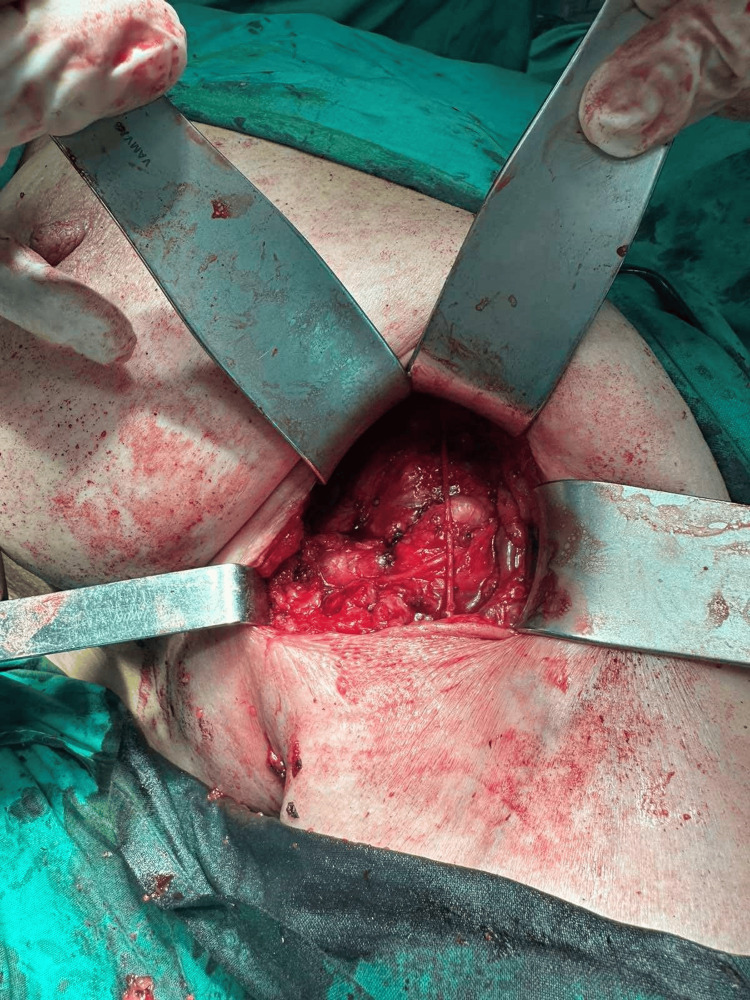
Intraoperative picture of the left axilla. Retractors provide optimal exposure of the tumor.

**Figure 4:**
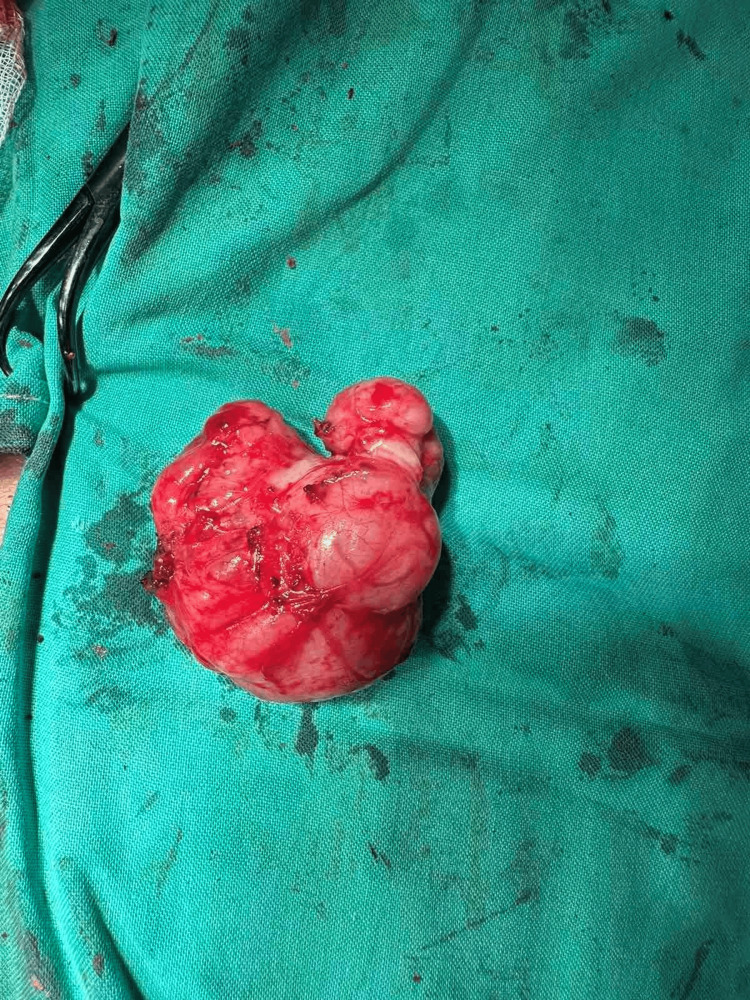
Partial excision of the tumor from the left axilla.

The histopathological report describes a lipomatous mass measuring 10 x 9 x 3.5 cm, within which 16 lymph nodes were found showing fatty degeneration and no neoplastic infiltration. The mass is composed of spindle cells with low cellularity, no atypia, and a myxoid stroma with sclerotic areas (Figures 5, 6).

**Figure 5:**
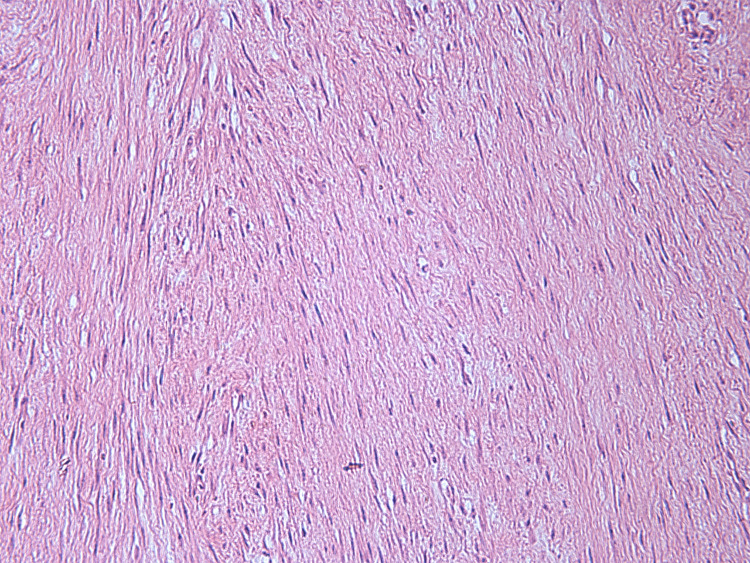
Histological examination of the lesion stained with hematoxylin and eosin (H&E) (original magnification ×20). Uniform spindle cells with pale eosinophilic cytoplasm arranged in intersecting fascicles. The cells lack nuclear hyperchromasia and cytological atypia. Thin-walled vessels with focal perivascular edema are evident.

**Figure 6:**
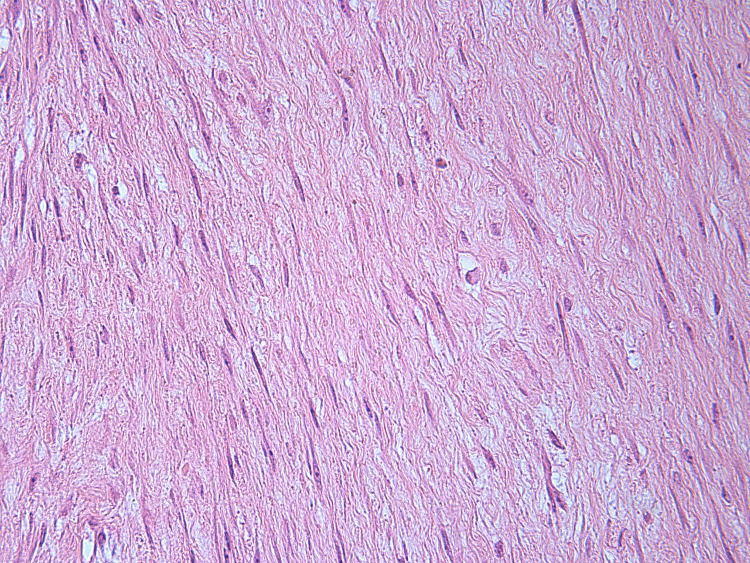
Histological examination of the lesion stained with hematoxylin and eosin (H&E) (original maginification x40). Uniform spindle cells with pale eosinophilic cytoplasm arranged in intersecting fascicles. The cells lack nuclear hyperchromasia and cytological atypia. Thin-walled vessels with focal perivascular edema are evident.

The immunohistochemical analysis revealed the following markers: β-catenin (nuclear staining positive), MUC4 (negative), SMA (focally positive), desmin (negative), S100 (negative), CD34 (negative), AE1/AE3 (negative), and EMA (negative). The Ki67 proliferation index is very low (<1%) (Figures 7, 8). The patient was advised to be referred to a specialized center for the treatment of desmoid tumors, where a multidisciplinary team would be available for her care. However, the patient declined further surgical treatment and did not return to the clinic.

**Figure 7:**
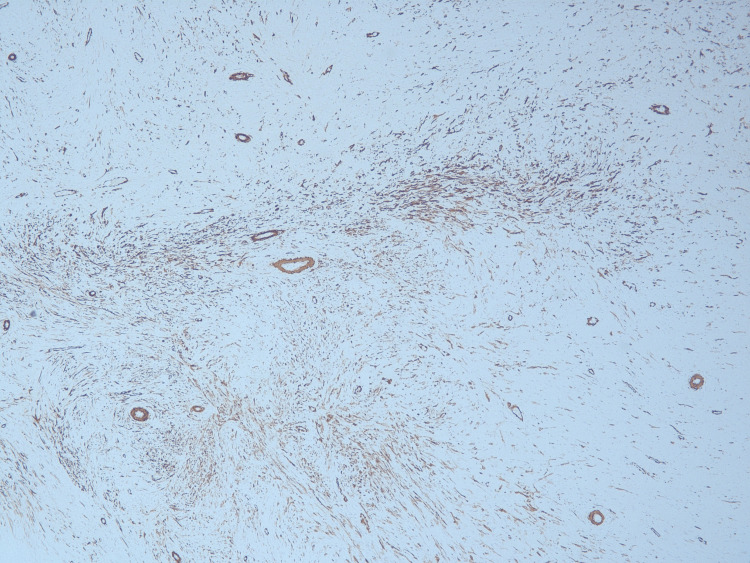
Partial cytoplasmic positivity for smooth muscle actin (SMA) in tumor cells, consistent with myofibroblastic differentiation.

**Figure 8:**
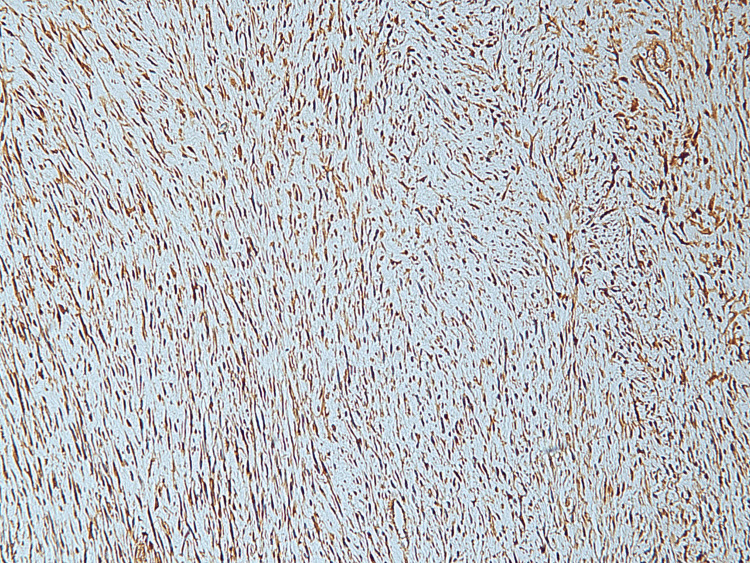
Immunohistochemical staining demonstrates nuclear β-catenin expression in lesional spindle cells, supporting the diagnosis of desmoid fibromatosis.

## Discussion

Desmoid tumors are primarily located in the mesentery or retroperitoneum, predominantly affecting young individuals. In 10% of cases, they appear as an extra-intestinal manifestation of Gardner's syndrome in patients with FAP [7]. These tumors do not typically metastasize but have high rates of local recurrence. They exhibit distinct histological features such as low cellularity, uniform spindle cells with small, distinct nuclei arranged in bundles. Their vascularization includes small arteries with veins exhibiting mild perivascular lymphatic infiltration. Their stroma is collagenous [8]. Cellular density, atypia, and atypical mitoses, which are indicators of malignancy, are not present.

Most desmoid tumors are attributed to disruptions in the WNT pathway. In FAP, they are due to mutations in the APC gene located on chromosome 5q21-22, which encodes a tumor-suppressing protein with complex and intricate actions. Deactivation of APC leads to increased translation and cellular proliferation. Most APC mutations associated with desmoid tumors are found at the 3' end of codon 1400 [9]. Some sporadic desmoid tumors arise from APC deactivation, but over 80% are APC wild-type with mutations in the CTNNB1 gene located on chromosome 13p22-p21.3. The CTNNB1 gene encodes β-catenin. Analysis of CTNNB1 mutations is usually not necessary, but in some cases, it helps distinguish between recurrent desmoid tumors and scar tissue. Whether specific CTNNB1 mutations assist in predicting desmoid tumor recurrence after surgical resection is not yet clarified, but a study by Domont et al. showed no significant difference in disease-free survival between individuals with CTNNB1 mutations, though it indicated worse outcomes in tumors with mutations compared to wild-type [10]. The common final pathway is β-catenin activation, and although immunohistochemistry is not necessary for diagnosis, nuclear β-catenin staining characterizes over 90% of desmoid tumors [8]. Immunohistochemistry for β-catenin is less reliable when material is obtained via fine needle aspiration (FNA). Immunoreactivity with CD117 has been reported in the literature; however, when appropriate conditions and methods are used, CD117 positivity is not observed [11].

There is controversy regarding the response to imatinib treatment, with some supporting its efficacy while others show it to have lower response rates compared to other systemic chemotherapy treatments [9,12]. Surgical resection remains the best therapeutic approach, but complete excision is often impossible due to extensive desmoid tumor infiltration, leading to recurrences of up to 38%. For these patients, systemic treatments such as anti-inflammatory drugs, hormonal therapy, chemotherapy, and radiation can be administered. In patients with FAP, ileal pouch surgeries can address the appearance of desmoid tumors [7].

## Conclusions

Desmoid tumor of the breast is a rare but manageable condition that requires individualized treatment approaches. It should always be considered in the differential diagnosis of recurrent breast mass or post-operative changes. Clinical examination helps to assess the size, location, and characteristics of any tumors. However, imaging studies and often a tissue biopsy are required to confirm the diagnosis of a desmoid tumor. MRI is usually the most useful imaging test, as it shows the relationship of the tumor to nearby muscles, nerves, and other structures. This information is significant for treatment planning. Additional tests may also be ordered to check for FAP if this genetic condition is suspected. This is important because it affects treatment decisions and recommendations for family screening. Treatment for desmoid tumors varies significantly depending on the size, location, symptoms, and growth rate of the tumor. The goal is to control the tumor while minimizing side effects and preserving quality of life. Many doctors in the literature prefer an initial ‘watchful waiting’ approach, especially for small asymptomatic tumors.
